# Effects of β-Carotin and Green Tea Powder Diets on Alleviating the Symptoms of Gouty Arthritis and Improving Gut Microbiota in C57BL/6 Mice

**DOI:** 10.3389/fmicb.2022.837182

**Published:** 2022-01-25

**Authors:** Yu Feng, Yanbo Yu, Zheng Chen, Lili Wang, Jingyu Ma, Xiaohui Bai, Yundong Sun, Dawei Wang

**Affiliations:** ^1^Department of Orthopedic, Shandong Provincial Hospital, Cheeloo College of Medicine, Shandong University, Jinan, China; ^2^Department of Orthopedic, Shandong Provincial Hospital Affiliated to Shandong First Medical University, Jinan, China; ^3^Department of Gastroenterology, Qilu Hospital, Shandong University, Jinan, China; ^4^Department of Clinical Laboratory, Shandong Provincial Hospital Affiliated to Shandong First Medical University, Jinan, China; ^5^Department of Microbiology, Key Laboratory for Experimental Teratology of Ministry of Education, Key Laboratory of Infection and Immunology of Shandong Province, School of Basic Medicine, Shandong University, Jinan, China

**Keywords:** β-carotin, green tea powder, gouty arthritis, gut microbiota, purine metabolism

## Abstract

As a chronic metabolic disease caused by disorders of purine metabolism, gout has shown increasing incidence rate worldwide. Considering that gout is not easily treated and cured, further studies are explored to prevent gout development through diet modification. Both β-carotin and green tea powder are rich in dietary fiber, which helps maintain the balance of gut microbiota in humans. The aim of this study was to investigate the effects of β-carotin and green tea powder diet on the prevention of gouty arthritis in relation to the bacterial structure of gut microbiota in mice. We successfully induced gouty arthritis in C57BL/6 mice by injecting monosodium urate (MSU) crystals and feeding high-fat diet (HFD), and further investigated the effects of additional β-carotin and green tea powder in the diets of mice on the prevention of gouty arthritis in mice. Our results showed that diet of β-carotin and green tea powder reduced the joint swelling and pain in mice with gout, reduced the levels of serum uric acid (UA) and three types of pro-inflammatory cytokines, i.e., interleukin-1β (IL-1β), interleukin-6 (IL-6), and tumor necrosis factor-α (TNF-α), improved the gut microbiota profile, and reduced the metabolic levels of purines and pyrimidines. In conclusion, our study provided evidence to support the application of β-carotin and green tea powder diet as a dietary adjustment method to prevent and treat gouty arthritis.

## Introduction

Gout is a chronic metabolic disease caused by disorders of purine metabolism (Kuo et al., 2015), characterized by elevated levels of uric acid (UA) in the blood to gradually form monosodium urate (MSU) crystals in the joints and soft tissues surrounding the joints, further inducing an acute inflammatory response with symptoms, such as fever, swelling, and burning and severe pains (Rees et al., 2014; So and Martinon, 2017). Studies have shown that the prevalence and incidence rates of gout are currently rapidly increasing worldwide (Kuo et al., 2015). For example, there are ~1.4 women and 4.0 men with gout per 1,000 people (Roddy and Doherty, 2010; Abhishek et al., 2017). Risk factors that have been identified for the development of gout include poor diet, hyperuricemia, and other metabolic syndromes (Roddy and Doherty, 2010). Both allopurinol and febuxostat are effective in the treatment of gout as the first-line agents to lower the level of uric acid (Abhishek et al., 2017). However, the use of these medicines has caused many common types of side effects in gout patients with negative impact on their health, such as rash, diarrhea, and abnormal hepatic functions (Wen et al., 2021). Therefore, prevention of gouty arthritis is extremely important and novel therapeutic treatments are needed to prevent the development of gouty arthritis.

Gut microbiota play significant roles in human health (Clemente et al., 2012). In recent years, a growing number of studies have revealed a strong connection between the development of gout and gut microbes (Guo et al., 2016; Shao et al., 2017). Furthermore, there is a growing body of evidence showing that the gut microbiota are also involved in purine metabolism and inflammatory responses induced by MSU crystals (Vieira et al., 2015; Chiaro et al., 2017). Moreover, a previous study suggested that the regulation of homeostasis in gut microbiota may be a potential therapeutic strategy of treating gouty arthritis with the traditional Chinese medicine “Simiao Decoction” (Lin et al., 2020a).

Dietary patterns affect the taxonomic compositions and biological functions of gut microbiota (Scott et al., 2013). For example, a high-fat diet (HFD) affects the composition of the gut microbiota in mice (Mullin, 2010) and significantly reduces the number of Bifidobacteria (Brinkworth et al., 2009). The short-chain fatty acids (SCFAs) play an important role in maintaining lipid homeostasis and reducing tissue inflammation, while different populations of gut microbes can modulate their beneficial effects by fermenting dietary fiber to produce SCFAs (Hills et al., 2019). Furthermore, studies have shown that green tea powder is rich in dietary fiber, which can further improve lipid metabolism in mice fed with HFD by altering the structure of gut microbiota (Wang et al., 2020). Moreover, the probiotic-containing yogurt has also been shown to significantly reduce the number of intestinal pathogens (Yang et al., 2012).

Consumption of dietary fiber and prebiotics that can be metabolized by microorganisms in the gastrointestinal tract is generally considered a dietary strategy to regulate the microbiota (Holscher, 2017). Both β-carotin and green tea powder are rich in dietary fiber (Wang et al., 2020; Matsumoto et al., 2021). Therefore, we hypothesized that the addition of these two substances to mouse feed could prevent gout development in mice by affecting the taxonomic components and functions of the gut microbiota in mice. In this study, we first established the mouse models of gouty arthritis induced by MSU crystal and HFD. We then further analyzed the taxonomic structure of gut microbiota and gout symptoms with the addition of β-carotin and green tea powder added either separately and in combination in the diet for mice. Our results showed that diets of β-carotin and green tea powder alleviated the symptoms of gouty arthritis in mice, reduced the levels of serum uric acid and three type of pro-inflammatory cytokines, i.e., interleukin-1β (IL-1β), interleukin-6 (IL-6), and tumor necrosis factor-α (TNF-α), improved the gut microbiota profile, and reduced the metabolic levels of purines and pyrimidines.

## Materials and Methods

### β-Carotin and Green Tea Powder

β-Carotin was purchased from Adamas Chemical Reagent Co., Ltd. (Shanghai, China, Cat. 1109142). Green tea “Longjing” was purchased from Hangzhou Zhenghao Tea Co. (Hangzhou, China). Green tea powder was made from green tea raw materials by hot water extraction, followed by dehydration and drying. The green tea ingredients were mixed in water at 90°C for 30 min and then the mixture was dewatered using a screw extruder at 0.5–1.2 MPa for 1 min, dried at 100–130°C for 3–5 h, and finally the dehydrated green tea leaves were ground into powder and stored at 4°C.

### Animals

Twenty-five specific pathogen-free (SPF) male C57BL/6 mice (4–6 weeks old with an average body weigher of 15 ± 3 g) were provided by Beijing Vital River Laboratory Animal Technology Co., Ltd. (Beijing, China) and housed in the SPF environment of Shandong Provincial Hospital Laboratory Animal Research Center. The experimental procedure was approved by the Laboratory Animal Management and Ethics Review Committee of Shandong University, China (Permit No.: MECSDUMS2012056). All mice were maintained under the standard environmental conditions (12/12 h light/dark cycle and 25 ± 1°C) with constant access to food and water.

### Dietary Treatments

After 1 week of rearing to adapt to the environment, all 25 mice were evenly and randomly divided into five groups, including the control group (CTL), the gouty arthritis model group (Model), the β-carotin diet group (Model + β-carotin), the green tea powder diet group (Model + GTP), and the β-carotin and green tea powder combined diet group (Model + Double). The mice in the CTL group were fed with a normal diet (containing 24% kcal from protein, 3.44 total kcal/g, and Beijing Keaoxieli Feed. Co., Ltd.) every day and were individually injected a total of 40 μl of phosphate buffered saline (PBS) into the right rear footpad once every 10 days. The other four groups of mice were fed daily with HFD (10% yeast extract) and were injected with MSU crystals (1 mg mixed in 40 μl PBS) into the right hind footpad once every 10 days (Lin et al., 2020b). The experiments lasted for 6 weeks after the mouse models of gouty arthritis were established. The proportion of green tea powder and β-carotin added to the feed was converted according to the daily weight of the oral feed. The mice in the Model + β-carotin and Model + GTP groups were supplemented with 0.05% β-carotin and 2% green tea powder in the HFD, respectively (Wang et al., 2020; Yang et al., 2020). The mice in the Model+Double group were fed with 2% green tea powder and 0.05% β-carotin added to the HFD.

### Sample Collection

Samples were collected at the end of the 6-week long experiments (i.e., 42 days). The mouse blood was collected through the orbital vein and then centrifuged at 1,300*g* and 4°C for 10 min. Subsequently, the mice were euthanized with CO_2_ with the fresh fecal materials collected immediately from the colon tissue of the mice and stored at −80°C. Simultaneously, the liver and foot joint tissues were collected, quickly frozen in liquid nitrogen, and then stored at −80°C.

### Preparation of the MSU Crystals

Monosodium urate crystals were prepared as described previously (Ruiz-Miyazawa et al., 2017). Briefly, the MSU (800 mg) was dissolved in boiling Milli-Q water (155 ml) and added with NaOH (5 ml), with the pH value adjusted to 7.2 using hydrochloric acid. The solution was cooled and centrifuged to collect the crystals, which were evaporated and stored after high temperature sterilization.

### Evaluation of Foot Joint Hypersensitivity and Oedema

A digital caliper (Meinaite, Germany) was used to measure the thickness of the footpads of each mouse before and 4, 24, 48, and 72 h after the injection of MSU crystals into the footpads of the mice. The degree of foot swelling in mice was calculated as the ratio of Δmm/mm (at zero time point) of the joints (Lin et al., 2020b). As described previously (Chaplan et al., 1994), von Frey filaments (UGO Basile, Italy) were used to measure the mechanical retreat threshold (MWT) to estimate the foot pain threshold of mice.

### Measurements of the Levels of Pro-inflammatory Cytokines and Enzymatic Activities

Thaw The samples of mouse foot joint and liver samples were thawed at room temperature and subsequently homogenized (0.05 g tissue per 1.0 ml buffer solution) and centrifuged for 10 min at 12,000 rpm and 4°C. The supernatants were assessed for the myeloperoxidase (MPO) activity in foot joint tissues and the xanthine oxidase (XOD) and adenosine deaminase (ADA) activities in hepatic tissues by following the manufacturer’s instructions (Jiancheng, Nanjing, China). Both XOD and ADA were enzymes involved in purine metabolism to promote the uric acid production (Lou et al., 2020). Three types of pro-inflammatory cytokines, i.e., IL-1β, IL-6, and TNF-α, were measured in serum and foot joint supernatant using the Mouse ELISA Commercial Kit (NOVUS, Germany) according to the manufacturer’s instructions of Varioskan Flash (Thermo Science, United States). The levels of serum UA in mice were measured by using TBA-40FR automated biochemical analyzer (Toshiba, Japan).

### Histopathological Assessment of Foot Joint

Foot joint tissues collected from mice were first rinsed with PBS, and then fixed overnight in 10% paraformaldehyde, followed by decalcification with EDTA for 3 weeks. The decalcified foot joint tissue was embedded in paraffin for sectioning. The sections with thickness of 5 μm were stained with hematoxylin and eosin (H&E) to evaluate the changes in the morphology and inflammation levels of the foot joint tissues.

### Gut Microbiota Analysis

Genomic DNA was extracted from the samples using the TIANamp Bacteria DNA Kit (Tiangen Biotechnology Co., Ltd., Beijing, China) according to the manufacturer’s instructions. Primers 27F and 1492R were used for PCR amplification (10 μl system, Solexa PCR) of the target area of 16S rRNA. The sequencing libraries were quality-checked, and the high-quality sequencing libraries were subjected to barcode identification and other necessary processes to obtain the circular co-sequencing (CCS) sequences. The optimized CCSs were clustered at the level of 97% similarity (USEARCH, version 10.0) with the species classification obtained based on the sequence composition of the operational taxonomic unit (OTU). The platforms 16S: The Silva database^1^ and RDP Classifier^2^ were used to analyze the species annotation and taxonomy as well as the diversity of gut microbiota. Alpha diversity analysis was performed to examine the species richness and diversity with the ACE, Chao1, Shannon, and Simpson indices for each sample calculated. The differences in the community composition and structure of different samples were compared through Beta diversity analysis, i.e., the principal coordinate analysis (PCoA). Finally, Metastats analysis was performed to compare the significant differences between the groups at the genus level, and the linear discriminant analysis effect size (LEfSe) analysis was used to screen the biomarkers with statistical differences between the groups (LDA score > 4). The “cor.test” function in the statistical software R was used to calculate the Spearman correlation coefficient between the microbial community and gout symptoms, visualized with a heat map (Li et al., 2020a). PICRUSt was used to infer the metabolic functions of the intestinal microbiota and to predict the molecular function of each sample based on the sequences of 16S rRNA-tagged genes (Langille et al., 2013). These predictions were pre-calculated for the annotation of genes based on the Kyoto Encyclopedia of Genes and Genomes (KEGG) database.^3^ The datasets presented in this study can be found in the National Center for Biotechnology Information (https://www.ncbi.nlm.nih.gov/sra/) database with the accession number PRJNA783957.

### Statistics

The biochemical data were analyzed and plotted using GraphPad Prism 8.0. The differences in gene expression among different groups were statistically evaluated based on *t*-test. Compositional differences in the microbiota between different groups were analyzed using the Kruskal–Wallis rank-sum test (*p* < 0.05).

## Results

### β-Carotin and Green Tea Powder Effectively Alleviate the Symptoms of Gout in Mice

**Figure 1:**
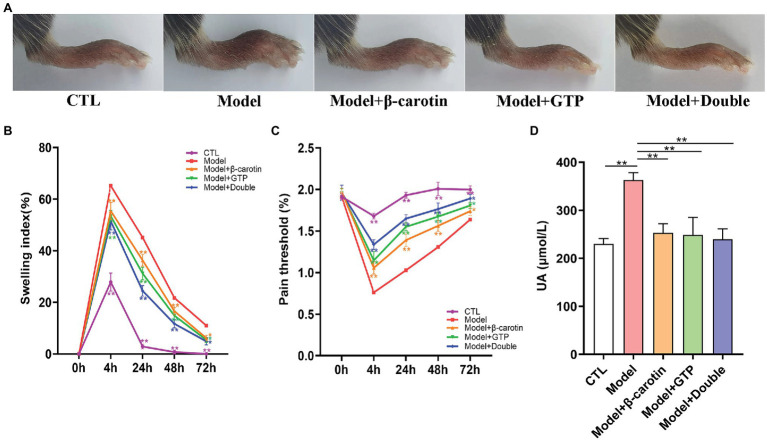
Effects of β-carotin and green tea powder on alleviating the symptoms of gout in mice. **(A)** Representative images of foot joints in five groups of mice. **(B)** Level of footpad swelling in five groups of mice. **(C)** Level of footpad pain threshold in five groups of mice. **(D)** Level of serum uric acid (UA) in five groups of mice. ^*^p < 0.05, ^**^p < 0.01.

The mouse model of gouty arthritis induced by treating the mice with MSU crystals and HFD was successful established. The levels of serum UA, the footpad swelling, and the pain threshold in mice were assessed to evaluate the effect of adding β-carotin and green tea powder to the diet on gout (Figure 1). Compared with the mice in the CTL group, the mice in the Model group showed evident foot joint swelling, with foot joint swelling decreased significantly after feeding mice with β-carotin and green tea powder (Figures 1A,B). Compared with the CTL group, the footpad mechanical pain threshold was reduced in the Model group mice, while addition of β-carotin and green tea powder alleviated the MSU-induced mechanical abnormalities in pain (Figure 1C). When mice were given a combination of both β-carotin and green tea powder, the most pronounced decrease in foot joint swelling and footpad mechanical pain threshold was observed (Figures 1B,C). Furthermore, feeding both β-carotin and green tea powder to mice in the Model + Double group or the separate feeding of β-carotin and green tea powder to the mice in the Model + β-carotin and Model + GTP group significantly decreased the level of serum UA compared to the Model group (Figure 1D).

### β-Carotin and Green Tea Powder Improves Gout-Related Inflammation Markers in Mice

**Figure 2:**
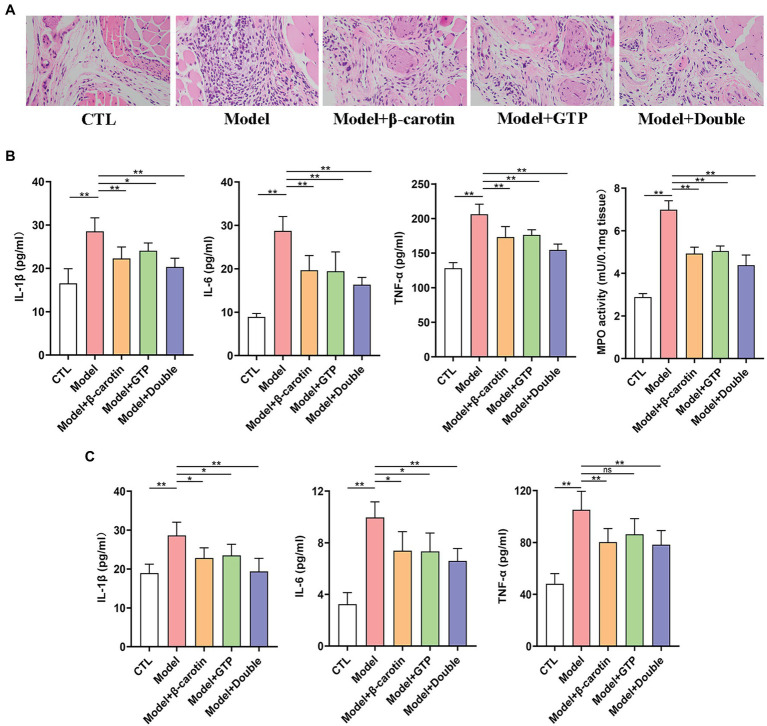
Effects of β-carotin and green tea powder on the improvement of gout-related inflammation markers in mice. **(A)** HE analysis of the footpad. **(B)** Level of cytokines [i.e., interleukin-1β (IL-1β), interleukin-6 (IL-6), and tumor necrosis factor-α (TNF-α)] and myeloperoxidase (MPO) activity in the foot joint tissues of five groups of mice based on ELISA. **(C)** Level of cytokines (i.e., IL-1β, IL-6, and TNF-α) in the serum of five groups of mice based on ELISA. ^*^p < 0.05, ^**^p < 0.01, and “ns” represents no statistical significance.

To further analyze the effects of β-carotin and green tea powder on gout, the inflammation of gout sites in different treatment groups of mice was evaluated (Figure 2). Results of the histological analysis showed that MSU crystals significantly increased the inflammatory cell infiltration in the foot joint compared to the CTL group, while feeding mice with β-carotin and green tea powder decreased the number of inflammatory cells in the foot joint (Figure 2A).

Previous studies have shown that IL-6 (Cavalcanti et al., 2016), IL-1β (So and Martinon, 2017), and TNF-α (Amaral et al., 2016) were associated with inflammatory activity in patients with gout. Compared with mice in the CTL group, the levels of IL-1β, IL-6, TNF-α, and MPO activity were significantly higher in the foot joints of mice in the Model group, showing that the administration of β-carotin and green tea powder significantly decreased the levels of these inflammatory markers (Figure 2B).

The expression levels of three pro-inflammatory cytokines (i.e., IL-1β, IL-6, and TNF-α) associated with gout were further investigated in serum (Figure 2C). Compared with the CTL group, the levels of IL-1β, IL-6, and TNF-α in serum were significantly increased in the Model group, but decreased in mice fed with β-carotin and green tea powder (Figure 2C).

### Effect of β-Carotin and Green Tea Powder on Purine Metabolism in Hepatic Tissue of Mice

**Figure 3:**
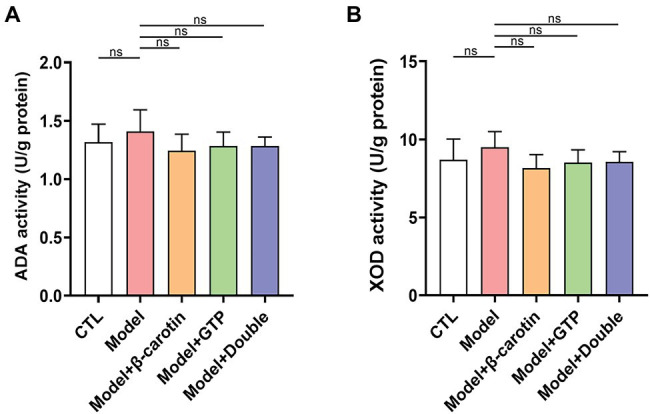
Effects of β-carotin and green tea powder on enzymatic activities related to purine metabolism in mouse hepatic tissue. **(A)** Level of adenosine deaminase (ADA) activity in the hepatic tissue of five groups of mice based on ELISA. **(B)** Level of xanthine oxidase (XOD) activity in the hepatic tissue of five groups of mice based on ELISA. “ns” represents no statistical significance.

The results of XOD and ADA activities measured in the hepatic tissues showed that both activities were not significantly elevated in the Model group compared with that of the CTL group (Figures 3A,B). It was noted that although feeding β-carotin and green tea powder to mice resulted in a decrease in the activities of both XOD and ADA, the differences were not statistically significant (Figures 3A,B).

### Alterations of Gut Microbiota

**Figure 4:**
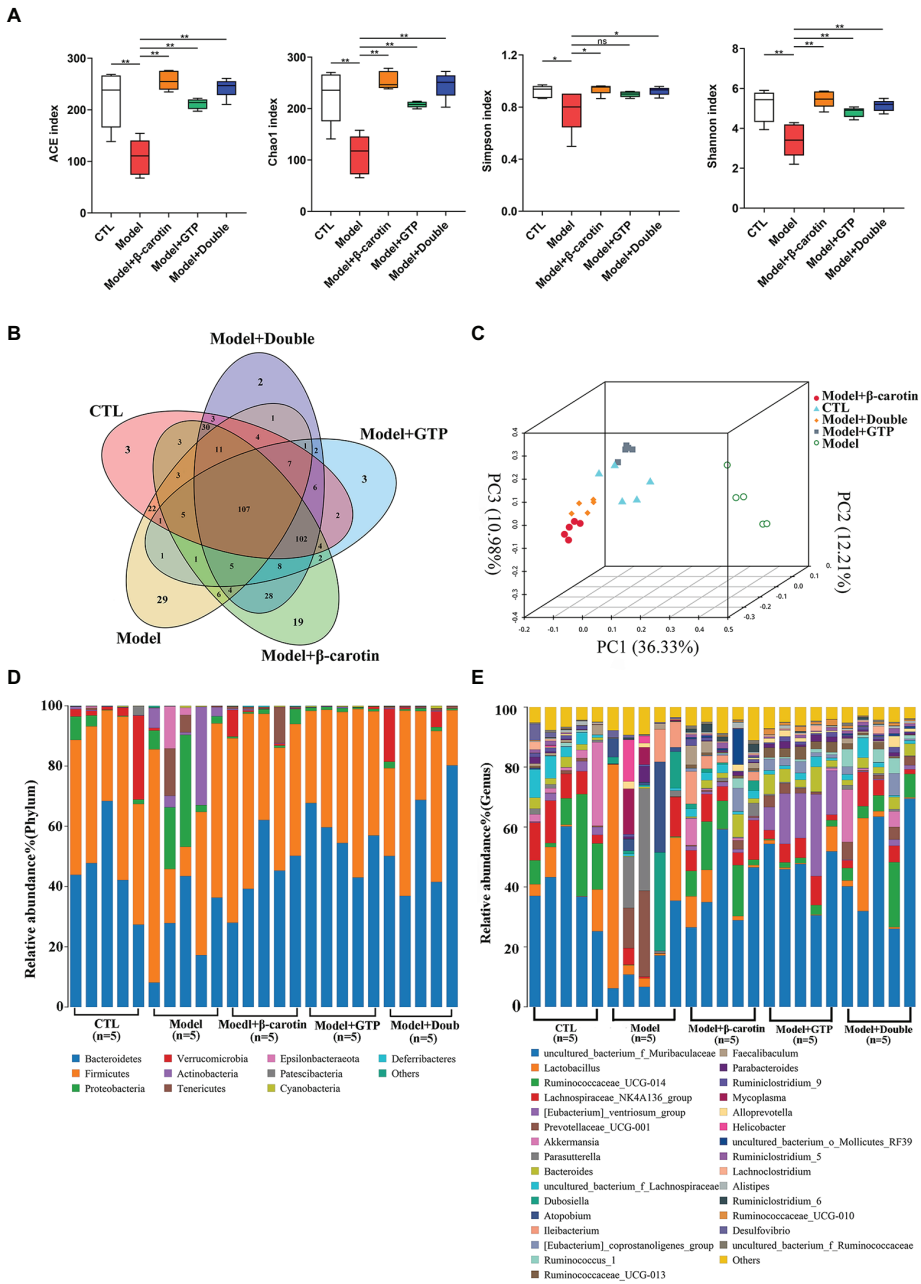
Alterations in gut microbiota among five groups of mice. **(A)** Alpha diversity analysis. **(B)** Venn diagram of operational taxonomic units (OTUs). **(C)** Principal coordinate analysis (PCoA) of the gut microbiome. **(D)** Composition of the gut microbiota at the phylum level among the five groups of mice. **(E)** Composition of the gut microbiota at the genus level among the five groups of mice. ^*^p < 0.05, ^**^p < 0.01, and “ns” indicates no statistical significance.

To investigate the effects of β-carotin and green tea powder on the taxonomic composition of gut microbiota in mice, we collected the fecal samples from mice for 16S rRNA sequencing analysis. The results showed that species richness based on the ACE and Chao1 indices was significantly decreased in mice with gout, while the microbial diversity as assessed by Shannon and Simpson indices showed a significant decrease in mice with gout (Figure 4A). The application of β-carotin and green tea powder normalized the ACE, Chao1, Shannon, and Simpson indices (Figure 4A). The analysis of OTUs in feces showed that there were a total of 107 OTUs among the five groups of mice, with 3, 29, 19, 3, and 2 OTUs unique to the CTL, the Model, the Model + β-carotin, the Model + GTP, and the Model + Double groups, respectively (Figure 4B). For the Beta diversity, the results of PCoA revealed significant differences in gut microbiota between the CTL and Model groups, while the gut microbiota of mice fed with β-carotin and green tea powder was significantly similar to that of mice in the CTL group (Figure 4C). At the phylum level, analysis of the composition of the gut microbiota in five groups of mice showed that Firmicutes and Bacteroidetes were the dominant bacterial taxa (Figure 4D). The relative abundance of Bacteroidetes was significantly lower in the Model group compared to the CTL group, and feeding mice with β-carotin and green tea powder normalized the abundance of Bacteroidetes (Figure 4D). At the genus level, the relative abundance of microorganisms, such as *Muribaculaceae*, *Ruminococcaceae_UCG-014*, and *Lachnospiraceae_NK4A136_group* was decreased in the Model group compared to the CTL group, and feeding mice with β-carotin and green tea powder increased the relative abundance of these microorganisms (Figure 4E).

**Figure 5:**
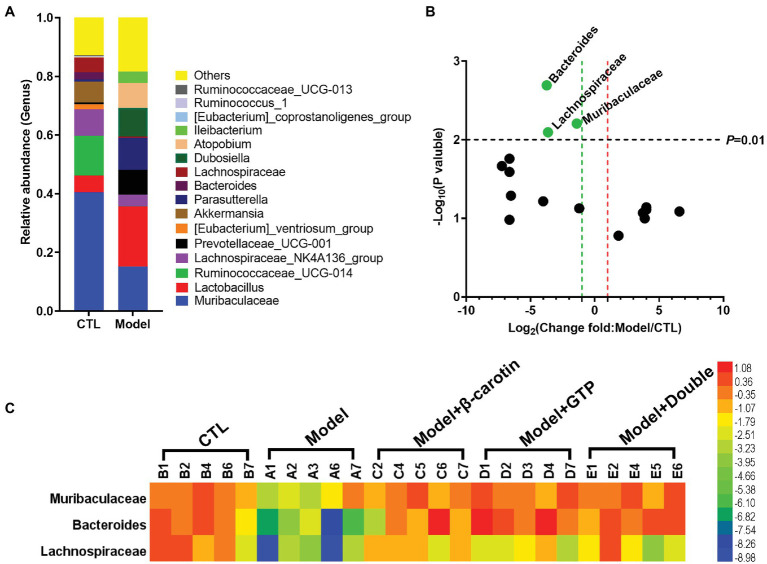
Comparative analysis of key bacteria at the genus level in five groups of mice. **(A)** Comparison of microbial differences between the mice in Model and control group (CTL) groups showing the microbial taxa with the average relative abundance >1%. **(B)** Volcano graph analysis of changes in the gut microbiota of mice in Model and CTL groups. The *p* value is based on the *t*-test. Bacteria with a relative abundance >1% in **(A)** are selected as key bacteria. **(C)** The heat map showing the relative abundance of key bacteria in each sample of the five groups of mice.

At the genus level, bacteria with relative abundance greater than 1% between the Model and CTL groups were compared to show that *Muribaculaceae*, *Lactobacillus*, *Ruminococcaceae_UCG-014*, *Lachnospiraceae_NK4A136_group*, *Akkermansia Parasutterella*, and *Prevotellaceae_UCG-001* were identified as the main taxa in the gut microbiota of both groups of mice (Figure 5A). Bacteria with relative abundance greater than 1% were presented in the volcano plots (Figure 5B). Compared with the CTL group, the relative abundance of *Muribaculaceae*, *Bacteroides*, and *Lachnospiraceae* in the Model group was significantly lowered (Figure 5B). A heat map showing the relative abundance of key bacteria in each sample was presented in Figure 5C. These results revealed that the relative abundance of *Muribaculaceae*, *Bacteroides*, and *Lachnospiraceae* was significantly decreased in the Model group compared to the CTL group, while feeding β-carotin and green tea powder to mice increased the relative abundance of these three taxa (Figure 5C).

### Interaction of Gut Microbiota

**Figure 6:**
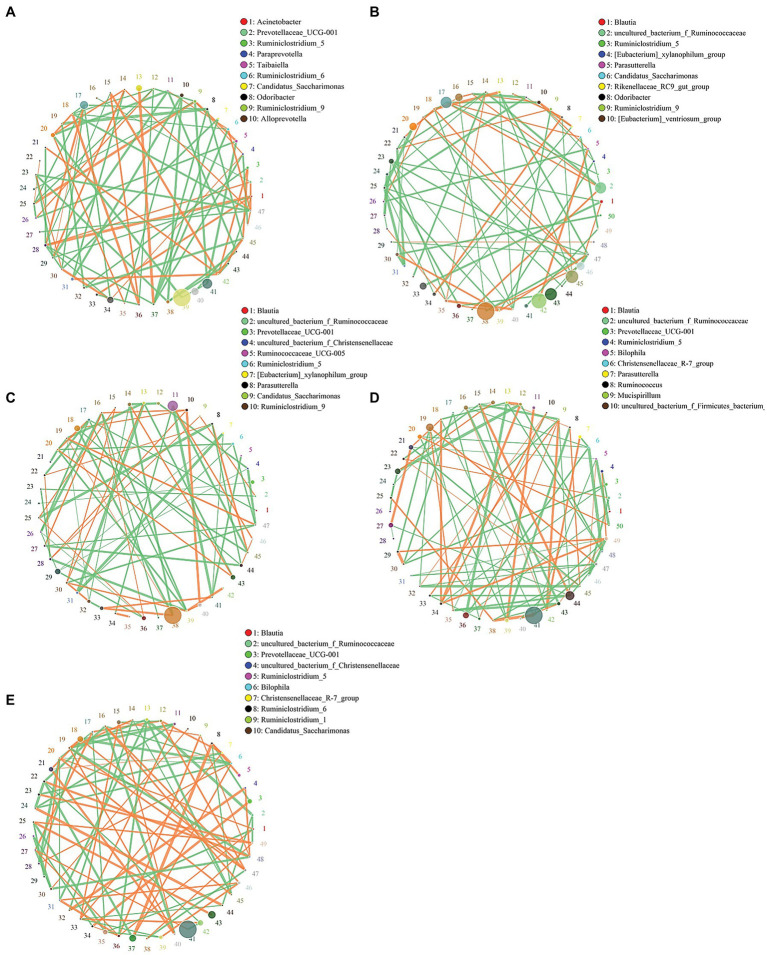
Correlation of microbiota at genus level in the intestines of five groups of mice, including the CTL group **(A)**, the Model group **(B)**, the Model + β-carotin group **(C)**, the Model + GTP group **(D)**, and the Model + Double group **(E)**. The size of the circle represents the relative abundance, the line represents the correlation between the two taxa at both end of the line, the thickness of the line represents the strength of the correlation, the orange line represents positive correlation, and the green line represents negative correlation.

Microorganisms with abundance TOP: 50 were selected to further investigate the correlation between gut microorganisms with the |Spearman correlation| ≥ 0.1 and *q* ≤ 0.05 set as the filtering parameters. Due to space constraints, only bacteria numbers 1–10 are listed. The results revealed strong interactions among gut microbes (Figure 6). The interactions among the intestinal microorganisms were reduced in the Model group of mice compared to the CTL group (Figures 6A,B), while feeding β-carotin and green tea powder to mice significantly increased the interactions between gut microbes (Figures 6C–E). For example, *Lachnoclostridium* (28) was positively correlated with both *Blautia* (1) and *Ruminococcaceae* (2) in the CTL group (Figure 6A). In the Model group, *Lachnoclostridium* (32) was only negatively correlated with *Ureaplasma* (14; Figure 6B), while in the Model + β-carotin group, *Lachnoclostridium* (30) interacted positively with *Candidatus_Soleaferrea* (29; Figure 6C). In the Model + GTP group, *Lachnoclostridium* (28) was positively correlated with *Candidatus_Saccharimonas* (9), *Ruminiclostridium_9* (10), and *Ruminiclostridium* (22; Figure 6D), whereas in the Model + Double group, *Lachnoclostridium* (30) interacted positively with *Candidatus_Saccharimonas* (10; Figure 6E). Furthermore, the most significant increase in the level of interaction between gut microbes in mice was observed after the addition of combined β-carotin and green tea powder to their diets, showing a predominantly positive correlation (Figure 6E).

### Metabolic Functions of the Gut Microbiota

**Figure 7:**
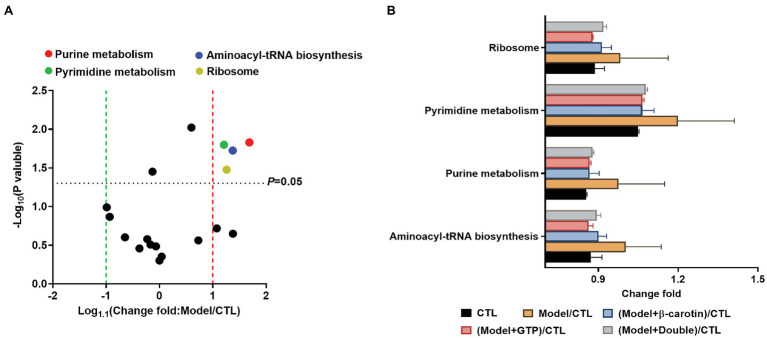
Comparison of the relative abundance of the functional profiles of the gut microbiota based on PICRUSt among the five groups of mice. **(A)** Volcano plot analysis of altered Kyoto Encyclopedia of Genes and Genomes (KEGG) pathways with an average relative abundance >1% between the mice in CTL and Model groups. The *p* value is based on a two-tailed and paired *t*-test. **(B)** Distinct gene categories selected based on significant differences in gene categories at level 2 (*t*-test, *p* < 0.05).

PICRUSt was used to predict the functions of gut microbiota based on 16S rRNA sequencing and the KEGG database (Figure 7). The results showed that compared with the CTL group, the levels of ribosomal synthesis, pyrimidine metabolism, purine metabolism, and aminoacyl-tRNA biosynthetic metabolic pathways of the gut microbiota in the mice of the model group were increased (Figure 7A), while feeding mice with β-carotin and green tea powder significantly reduced the activities of these metabolic pathways (Figure 7B).

### Correlation Between Key Gut Microbes and Pro-inflammatory Cytokines, Metabolites, and Metabolic Pathways in Mice

**Figure 8:**
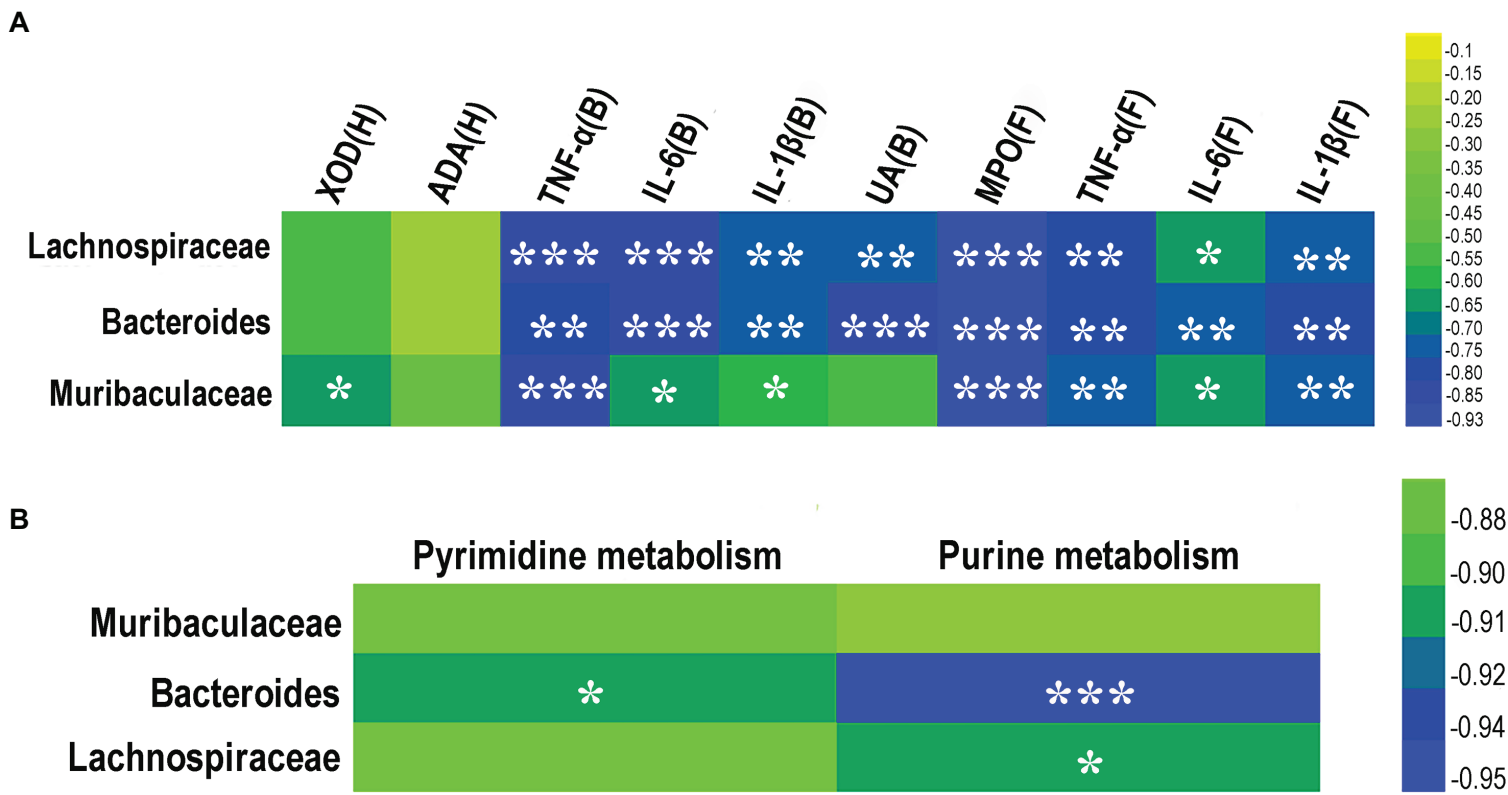
Correlation between key gut microbes and pro-inflammatory cytokines, enzymes, and metabolic pathways from three taxa of gut microbes (i.e., *Lachnospiraceae*, *Bacteroides*, and *Muribaculaceae*). **(A)** Correlation among pro-inflammatory cytokines and enzymes. **(B)** Correlation between purine and pyrimidine metabolic pathways. The color of the cell indicates the relative *R* values ranging from −0.95 to 0 (i.e., blue to green). B, blood; H, hepatic tissue; and F, foot joint tissues. ^*^p < 0.05, ^**^p < 0.01, and ^***^p < 0.001.

A heat map analysis of Spearman rank correlation coefficients was performed to determine the correlations between key bacterial taxa and the levels of IL-1β, IL-6, TNF-α, UA, activities of MPO, XOD, and ADA, and purine and pyrimidine metabolisms (Figure 8). The results showed that *Bacteroides* and *Lachnospiraceae* were negatively correlated with the levels of IL-1β, IL-6, TNF-α, UA, and MPO activity, but not significantly correlated with the levels of XOD and ADA activities (Figure 8A). *Muribaculaceae* was negatively correlated with the levels of IL-1β, IL-6, TNF-α, and the activities of MPO and XOD, but not significantly correlated with the levels of ADA activity and UA (Figure 8A). *Bacteroides* was negatively correlated with both pyrimidine and purine metabolisms, *Lachnospiraceae* was negatively correlated with purine metabolism (Figure 8B), while *Muribaculaceae* was not significantly correlated with either pyrimidine metabolism or purine metabolism (Figure 8B).

## Discussion

With the increased prevalence of gout risk factors, such as obesity, hypertension, kidney disease, and hyperlipidemia (Elfishawi et al., 2018), the incidence, hospitalization, and health care costs related to gout have increased significantly and the gout has emerged as a public health problem, causing severe socioeconomic burden (Kuehn, 2018). However, the traditional treatments of gout are generally ineffective with severe side effects. Therefore, it is extremely important to identify novel strategies to prevent the development of gouty arthritis. In the present study, our results showed that the addition of β-carotin and green tea powder to the diets of mice effectively improved the symptoms of gout induced by the combination of MSU crystals and HFD and improved the gut microbiota profile of mice with gout.

Green tea powder contains a variety of nutritional and functional components, including phenols, catechins, and dietary fiber (Onakpoya et al., 2014; Wang et al., 2020), which not only prevents tooth decay, but also reduces the absorption of cholesterol and lipids in the gastrointestinal tract (Koo and Cho, 2004). The polyphenolic compounds in green tea have the effect of scavenging oxygen and nitrogen free radicals, and can also reduce serum UA concentration by regulating urinary UA excretion in mice with hyperuricemia (Frei and Higdon, 2003; Chen et al., 2015). In addition, the catechins in green tea have the potential to increase UA excretion (Kawakami et al., 2021). Numerous studies have shown that dietary fiber promotes the growth of beneficial microbes and inhibits the development of harmful microbes, helping maintain the balance of gut microbiota (Wang et al., 2020). As one type of important natural pigment in plants with multiple physiological functions (Wang et al., 2017), β-carotin is generally considered a safe form of vitamin A due to its highly-regulated intestinal absorption (Shete and Quadro, 2013). Studies have shown that β-carotin acts mainly as a type of effective antioxidant in scavenging peroxyl radicals (Jomova and Valko, 2013).

Gout is a disease with the disorders of purine metabolism involved in its development (Kuo et al., 2015), while the gut microbiota play an important role in purine metabolism (Chiaro et al., 2017). Furthermore, the gut microbiota play numerous functions in the development of the immune system and in maintaining the integrity of the intestinal barrier (O'Hara and Shanahan, 2006). Healthy gut microbiota show an important impact on the overall health of the host, while the alterations in the gut microbial community lead to disease susceptibility (Compare et al., 2016). Studies have shown that the gut microbiota is involved in the response to MSU crystals in a gout model of mice, whereas the germ-free mice or mice treated with antibiotics do not respond to the injections of MSU crystals (Vieira et al., 2015). Based on the 16S rRNA sequencing (Lin et al., 2020b), the top three relatively dominant phyla of the mouse gut microbiota were Firmicutes, Bacteroidetes, and Proteobacteria. In our study, the relatively dominant phyla of the mouse gut microbiota were Firmicutes and Bacteroidetes, while the MSU crystals and HFD altered the composition of the gut microbiota mainly by decreasing the relative abundance of Bacteroidetes. At the genus level, MSU crystals and HFD significantly reduced the relative abundance of *Muribaculaceae*, *Bacteroides*, and *Lachnospiraceae*.

Studies have shown that members of *Lachnospiraceae* are the main producers of butyrate (Wen et al., 2021). Furthermore, studies have revealed the anti-inflammatory effects of butyrate by inhibiting class I histone deacetylase and MSU-induced cytokine production in patients with gout (Cleophas et al., 2016, 2017). Previous studies have shown that members of *Lachnospiraceae* associated with the production of SCFAs are depleted in patients with gout (Shao et al., 2017). Moreover, members of *Lachnospiraceae* can prevent human colon cancer by producing butyric acid (Langille et al., 2014). In this study, similar results were revealed showing the decreased relative abundance of *Lachnospiraceae* and the significantly negative correlation between the relative abundance of *Lachnospiraceae* and the levels of UA, IL-1β, IL-6, TNF-α, MPO activity, and purine metabolism in mice with gout.

*Muribaculaceae* are the dominant microbiota in the mouse gut (Seedorf et al., 2014) and are closely associated with the production of SCFAs (Wen et al., 2021). Studies have shown that genes involved in carbohydrate metabolism are upregulated in members of *Muribaculaceae* (Chung et al., 2020). Recent studies have reported that HFDs decrease the relative abundance of *Muribaculaceae* in the fecal samples of mice (Mu et al., 2020). In our study, injection of MSU crystals and feeding with HFD resulted in a decrease in the relative abundance of *Muribaculaceae* in the gut microbiota with a negative correlation observed between *Muribaculaceae* and the levels of IL-1β, IL-6, TNF-α, and XOD and MPO activities, whereas no association was observed between the relative abundance of *Muribaculaceae* and purine metabolism, which is closely related to the development of gout.

*Bacteroides* are the common and abundant bacterial components of the gut microbiota that maintain complex and generally beneficial relationships with their hosts (Wexler, 2007). *Bacteroides* can digest glycans derived from plants and hosts to produce healthy metazoans (Singh, 2019). Previous studies have shown that primary gout is closely associated with the overall alteration in intestinal *Bacteroides* (Xing et al., 2015), while an impaired jejunal intestinal barrier and a significant reduction in the amount of *Bacteroides* at the genus level were revealed in geese with gout (Ma et al., 2021). In our study, injection of MSU crystals and feeding with HFD caused a decrease in the relative abundance of *Bacteroides*, which was significantly negatively correlated with the levels of UA, IL-1β, IL-6, TNF-α, MPO activity, and purine and pyrimidine metabolisms in mice with gout. Therefore, it is speculated that both *Bacteroides* and *Lachnospiraceae* may promote gout production by affecting purine metabolism.

The balance of microbial interactions plays an important role in maintaining tissue homeostasis and human health (Li et al., 2020b). For example, *Lactococcus lactis* produces the lactic acid streptococcal peptides that inhibit pathogens such as *Staphylococcus aureus* through population sensing of their own populations (Abisado et al., 2018). In our study, results showed that combined feeding of β-carotin and green tea powder to mice with gout significantly increased the interactions between gut microbes, with predominantly positive correlations. These positive effects may play a positive role in alleviating the gout symptoms. It is noted that further studies are necessary to verify the findings revealed in our study. In addition, we have observed that combined feeding of β-carotin and green tea powder to mice with gout did not achieve our expected goal of preventing gout. This suggests that the combination of the two has no synergistic effect on the prevention of gout, but our study shows that the combination of the two enhanced the interaction of gut microbiota, especially the positive effect.

Furthermore, the activities of ADA and XOD were evaluated in the hepatic tissue of mice to further investigate the mechanism underlying the decreased purine metabolism. The results showed that feeding both the β-carotin and green tea powder reduced the activities of ADA and XOD, but the difference was not statistically significant, probably due to the insufficient treatment time. Although the reduction of ADA and XOD activity levels is not significant, we have observed that the gout symptoms of mice have been significantly improved. It may be that β-carotin and green tea powder reduce the serum uric acid level by affecting the gut microbiota, thereby further improving the symptoms of gout.

## Conclusion

In conclusion, our results suggested that both β-carotin and green tea powder were effective in alleviating the inflammatory response to gout induced by the MSU crystals and feeding of HFD and improving the gut microbiota structure in mice with gout. Our study provides the strong experimental evidence to support the application of both β-carotin and green tea powder diet as a dietary adjustment strategy to prevent gouty arthritis in mice.

## Data Availability Statement

The datasets presented in this study can be found in online repositories. The names of the repository/repositories and accession number(s) can be found in the article/supplementary material.

## Ethics Statement

The animal study was reviewed and approved by China Council for Animal Care and Utilization Committee of Shandong University, China.

## Author Contributions

DW conceived this study. YF and YY collected the data and drafted the manuscript. XB, YS, ZC, LW, and JM carried out the experiments and performed data analyses. All authors contributed to the article and approved the submitted version.

## Funding

This study was financially supported by the National Natural Science Foundation of China (Grant/Award Numbers: 81972057, 81670942, 82070540, and 82172313), the Special Funds for Taishan Scholar Project (Grant/Award Number: tsqn202103180), and the Major Innovation Project of Shandong Province (Grant/Award Number: 2021GXGC011305).

## Conflict of Interest

The authors declare that the research was conducted in the absence of any commercial or financial relationships that could be construed as a potential conflict of interest.

## Publisher’s Note

All claims expressed in this article are solely those of the authors and do not necessarily represent those of their affiliated organizations, or those of the publisher, the editors and the reviewers. Any product that may be evaluated in this article, or claim that may be made by its manufacturer, is not guaranteed or endorsed by the publisher.

## Footnotes

^1^ https://www.arb-silva.de/

^2^ https://www.findbestopensource.com/product/rdp-classifier

^3^ https://www.genome.jp/kegg/
